# Questions about complementary and alternative medicine to the Regional Medicines Information and Pharmacovigilance Centres in Norway (RELIS): a descriptive pilot study

**DOI:** 10.1186/1472-6882-14-56

**Published:** 2014-02-14

**Authors:** Jan Schjøtt, Hilde Erdal

**Affiliations:** 1 Section of Clinical Pharmacology, Laboratory of Clinical Biochemistry, Haukeland University Hospital, 5021 Bergen, Norway; 2 Institute of Clinical Science, Faculty of Medicine and Dentistry, University of Bergen, Bergen, Norway; 3 Regional Medicines Information and Pharmacovigilance Centre (RELIS Vest), Haukeland University Hospital, Bergen, Norway

**Keywords:** Drug information, Complementary and alternative medicine, Decision support

## Abstract

**Background:**

Provision of clinically relevant information about complementary and alternative medicine (CAM) to health care professionals is not well described. The aim of the study was to assess questions about CAM to the Regional Medicines Information and Pharmacovigilance Centres in Norway (RELIS).

**Methods:**

All question-answers pairs (QAPs) in the RELIS database indexed with alternative medicine from 2005-2010 constituted the study material. A randomly selected sample of 100 QAPs was characterized with regard to type of question (category, patient-specific or general), occupation and workplace of enquirer, the type of information search performed (simple or advanced), and if the answers contained information to provide factual or consultative replies (facts about or advice on clinical use of CAM, respectively). Proportions were compared with Fisher’s exact test with significance at the 0.05 level.

**Results:**

One thousand and thirty-eight (7.7%) out of 13 482 questions involved CAM. Eighty-two out of 100 questions concerned products containing one or more herbs, vitamins and minerals as well as other substances. Thirty-eight out of 100 questions concerned the category documentation (substance identification and/or literature reports about clinical effects), 36 interactions, 16 adverse effects, 9 pregnancy and lactation, and 1 question concerned contraindications. Sixty-three questions were patient-specific and 37 general. Fifty-four questions came from physicians, 33 from pharmacists and 13 from others (including nurses, midwives, students, CAM practitioners, and the public). Pharmacists asked more frequently about interactions while physicians asked more frequently about adverse effects (p < 0.05). Seventy-six of the questions came from outside hospital, mainly general practice and community pharmacies. Fifty-nine answers were based on a simple and 41 on an advanced information search. Thirty-three factual and 38 consultative answers were provided. In 29 answers, search provided no information. Lack of information to provide an answer was not significantly different between patient-specific (31.7%) and general questions (24.3%).

**Conclusions:**

General practice and community pharmacies are the main sources for questions about CAM to RELIS. Physicians are concerned about adverse effects while pharmacists are concerned about interactions. Lack of information to provide answers to patient-specific and general questions about CAM represents a problem.

## Background

RELIS is a network of four regional medicines information and pharmacovigilance centres. The centres are localised at university hospitals in Norway, where pharmacists and clinical pharmacologists answer questions from health care professionals (mainly physicians and pharmacists) working in hospitals, hospital pharmacies, general practice and community pharmacies [1].

During the last decade, the proportion of questions involving complementary and alternative medicine (CAM) to RELIS has been about 8%. In 2005, 151 questions among 1876 involved CAM compared to 189 among 2420 in 2010. This reflects the use of this type of medicine both for acute symptoms, but also chronic diseases among the public. The working method in RELIS is to find evidence-based information and relate it to the clinical setting and individual characteristics of a patient (problem-oriented drug information). Thus, questions from health care professionals about CAM typically concern patients with chronic diseases where alternative medicine is added to an established drug therapy and decision support is requested. An example of a typical question to RELIS concerned a patient with ovarian cancer with liver metastases where treatment with liposomal doxorubicin was planned. The patient used CAM including *Taraxacum officinale* (dandelion), *Curcuma longa* (turmeric), and *Silybum marianum* (milk thistle). The hospital physician asked if CAM interacted with doxorubicin or interfered with the metastatic cancer disease. Thus, questions to RELIS frequently represent the most complex drug-related clinical problems where other drug information sources are either inconsistent or insufficient to solve them. The time used to answer questions about drugs in RELIS is dependent on the type of literature search that has to be performed [2]. Availability of high quality evidence-based databases is of major importance to facilitate literature searches, and this is also of importance regarding questions about CAM [3,4].

The growing interest for CAM in the public [5] is also reflected in the conventional health care system, which RELIS serve. CAM is presently offered in about 50% of Norwegian hospitals compared to one-third of Danish hospitals [6]. Furthermore, a high percentage of herbal co-use among patients using conventional drugs in general practice has been shown in a Norwegian study. In this study, three out of four patients did not discuss herbal use with any health care professional [7]. An Australian study found that the current lack of knowledge about CAM among general practitioners (GPs) hampered communication with patients [8]. Education and information needs among both health care students and professionals concerning CAM have been reported in European studies as well [9,10]. Our experience with queries about CAM to RELIS in Norway supports this perception. The questions often include statements that the enquirer needs information and advice due to lack of knowledge and competence about the subject.

We found sparse documentation of problem-oriented drug information and need of decision support associated with CAM in the literature, and believe the present pilot study could identify some challenges and areas of improvement for medicines (or drug) information centres.

## Methods

### Question-answer pairs (QAPs)

The RELIS database is a full-text, QAP database shared by RELIS where QAPs from each region of Norway are indexed and published to be searchable (in Norwegian) for health care professionals free of charge [1]. All QAPs in the RELIS database indexed with alternative medicine (the term for CAM) from 2005-2010 constituted the study material. A randomly selected sample of 100 QAPs was collected with use of Research Randomizer (http://www.randomizer.org). Demographics of each enquirer (occupation, workplace) are registered in the database. Furthermore, data is recorded on whether the question is general or patient-specific, and if it concerns mainly an interaction, adverse effects, pregnancy, documentation or other predefined categories. The category documentation involves questions about substance identification and/or literature reports about clinical effects. The drugs involved in a QAP are registered according to their generic name, trade name and ATC-number according to the Anatomical Therapeutic Chemical (ATC) classification developed by the World Health Organization [11]. The text and references in each QAP was examined by HE. In case of any questions about classification, JS was consulted for a final agreement.

### Analysis of the questions

The questions were characterized with regard to type of category (e.g. adverse effects, interactions, pregnancy), occupation and workplace of enquirer, if the questions were patient-specific (concerned a particular patient) or general (concerned guidelines for clinical practice or a population of patients or based on academic interest). In addition the number and category of CAM, and number of drugs in the questions were described.

### Analysis of the answers

The type of information search performed was based on the type of references and description of search strategy in the text of the answers. Categories of information search were based on a previous publication from RELIS [2]. If it was necessary to search the RELIS database, databases containing monographs like the Micromedex, the summary of product characteristics (SPC) for the drug, Natural Medicines Comprehensive Database, Stockley’s Herbal Medicines Interactions, reference books and/or colleagues/other health care professionals only, the search was categorised as simple. If searches in databases like Medline, Embase or Cochrane to obtain original articles were necessary or additional information was needed or not available as a result of an extensive the search, the search was categorised as advanced. The answers were characterized with regard to if they contained information to provide factual or consultative replies according to a definition by Davies et al. [12]. Factual answers, such as the therapeutic dose of a drug (or substance) or its half-life, or synonyms for traditional herbal medicines, can usually be located in textbooks, monographs or databases. Answers that included clinical advice on a specific case and entailed communication with a health care professional on the possible benefits and hazards of one or more courses of action were called consultative.

### Statistics

The data were analysed using SPSS 17.0 (SPSS Inc., Chicago IL). The Fisher’s exact test was applied when comparing categorical variables. To account for multiple comparisons (z-test), the significance level was adjusted using the Bonferroni method. P values < 0.05 were considered significant.

## Results

### Analysis of the questions

In the period from 2005-2010, RELIS received a total of 13 482 questions, where 1038 (7.7%) involved CAM. Fifty-four out of 100 enquirers were physicians, 33 pharmacists and 13 others (including nurses, midwives, students, CAM practitioners and the public) (Table 1). Thirty-five physicians were general practitioners, 3 worked in private practice and 16 in hospital. Twenty-five pharmacists worked in community pharmacies, 7 in hospital pharmacies and 1 in another type of institution. Nine questions concerned herbal medicines, 5 vitamins and minerals, 68 natural medicines with several ingredients including herbs, vitamins and minerals as well as other substances, and 7 were categorized as others (e.g. homeopathy, acupuncture, and alkaline water). Eleven questions concerned questions about two (n = 9) or three (n = 2) concomitant categories of CAM. The number of substances in the questions ranged between 1 and 21, sixty-five concerned one substance, 12 two, and 23 three or more. The number of drugs in the questions ranged between 0 and 10. Fifty-two concerned no drug, 25 one, 7 two, and 16 three or more. The three most common categories of questions concerned documentation (n = 38), interactions (n = 36) and adverse effects (n = 19), while 9 concerned pregnancy and lactation and 1 contraindications. Sixty-three questions were patient-specific while 37 were categorised to be more general. There were no significant differences between the two major occupations physicians and pharmacists with regard to category of CAM, number of substances or drugs in the questions. Seventy-six of the questions came from outside hospital and hospital pharmacies (mainly from general practice or community pharmacies). Patient-specific questions were more frequent than general questions in hospitals, and among physicians (all comparisons, p < 0.05). Table 2 shows that pharmacists asked more frequently about interactions while physicians asked more frequently about adverse effects (all comparisons, p < 0.05).

**Table 1:** Occupation and workplace of enquirers (N = 100) about CAM to RELIS

	**Workplace**					
**Occupation**	**General practice (N = 36) fraction %**	**Hospital (N = 17) fraction %**	**Community pharmacy (N = 26) fraction %**	**Hospital pharmacy (N = 7) fraction %**	**Private or community practice (N = 8)**^**b **^**fraction %**	**Other (N = 6) fraction %**
Physicians (N = 54)	35/54 64.8	16/54 29.6			3/54 5.6	
Pharmacists (N = 33)			25/33 75.8	7/33 21.2		1/33 3.0
Midwifes (N = 3)					3/3 100.0	
Nurses (N = 2)	1/2 50.0	1/2 50.0				
Other (N = 8)^a^			1/8 12.5		2/8 25.0	5/8 62.5

CAM = Complementary and alternative medicine; RELIS = Regional Medicines Information and Pharmacovigilance Centres in Norway.

^a^Including 1 pharmacy student, 1 acupuncturist, 1 homeopath, 1 employed in public child care, and 4 public requests (patient or private person).

^b^Including private practice for physicians and CAM practitioners, and community practice for midwifes.

**Table 2:** Category of questions by occupation (N = 100) about CAM to RELIS

	**Category**				
**Occupation**	**Documentation (N = 38) fraction %**	**Interactions (N = 36) fraction %**	**Adverse effects (N = 16) fraction %**	**Pregnancy and breastfeeding (N = 9) fraction %**	**Contraindications (N = 1) fraction %**
Physicians (N = 54)	23/54 42.6	15/54 27.8	12/54 22.2*	3/54 5.6	1/54 1.9
Pharmacists (N = 33)	10/33 30.3	20/33 60.6**	1/33 3.0	2/33 6.1	
Midwifes (N = 3)				3/3 100.0	
Nurses (N = 2)	1/2 50.0			1/2 50.0	
Other (N = 8)^a^	4/8 50.0	1/8 12.5	3/8 37.5		

CAM = Complementary and alternative medicine; RELIS = Regional Medicines Information and Pharmacovigilance Centres in Norway.

^a^Including 1 pharmacy student, 1 acupuncturist, 1 homeopath, 1 employed in public child care, and 4 public requests (patient or private person).

*Significantly more questions among physicians compared to pharmacists, p < 0.05.

**Significantly more questions among pharmacists compared to physicians, p < 0.05.

### Analysis of the answers

While monographs and databases were cited in 59 of the answers (simple search) advanced search strategies was used in 41. Three answers did not have references. Table 3 shows simple or advanced search strategies according to references or text in the 100 answers. Table 4 shows if the answers contained information to provide consultative or factual replies to 100 patient-specific or general questions. Lack of information was found in 24.3% of the answers to general questions and in 31.7% of the answers to patient-specific questions. The corresponding figures (lack of information) were 31.2% for adverse effects, 33.3% for pregnancy and breastfeeding, and one answer to the single question about contraindications (100%). Answers to questions about interactions lacked information significantly more frequently than answers to questions about documentation (44.4% versus 10.5%, p < 0.05). There were no significant differences between the two major occupations physicians and pharmacists with regard to search strategies or information found to provide answers.

**Table 3:** Search strategy and references in answers to questions (N = 100) about CAM to RELIS

**Simple search**^**a **^**(N = 59)**		**Advanced search**^**b **^**(N = 41)**	
**Previous answers from the RELIS database fraction %**	**Monographs and databases fraction %**	**Original articles fraction %**	**Additional information needed or not available fraction %**
13/59 22.0	46/59 78.0	32/41 78.0	9/41 22.0

CAM = Complementary and alternative medicine; RELIS = Regional Medicines Information and Pharmacovigilance Centres in Norway.

^a^Simple search includes the RELIS database, databases containing monographs like the Micromedex,Stockley’s Herbal Medicines Interactions, the summary of product characteristics (SPC) for a drug, Natural Medicines Comprehensive Database, reference books and/or colleagues/other health care professionals only.

^b^Advanced search include databases like Medline, Embase or Cochrane to obtain original articles if necessary or additional information needed or not available.

**Table 4:** Information in the answers to questions (N = 100) about CAM to RELIS

**General questions (N = 37)**^**a**^			**Patient-specific questions (N = 63)**^**b**^		
**Information available for factual answers**^**c **^**fraction %**	**Information available for consultative answers**^**d **^**fraction %**	**No information available for either factual or consultative answers fraction %**	**Information available for factual answers**^**c **^**fraction %**	**Information available for consultative answers**^**d **^**fraction %**	**No information available for either factual or consultative answers fraction %**
21/37 56.8*	7/37 18.9	9/37 24.3	12/63 19.0	31/63 49.2**	20/63 31.7

CAM = Complementary and alternative medicine; RELIS = Regional Medicines Information and Pharmacovigilance Centres in Norway.

*Significantly more among general questions, p < 0.05.

**Significantly more among patient-specific questions, p < 0.05.

^a^General questions concern guidelines for clinical practice or a population of patients or based on academic interest.

^b^Patient-specific question concerned a particular patient.

^c^Answers like the therapeutic dose of a drug or its half-life or synonyms for traditional herbal medicines which can usually be located in textbooks, monographs or databases.

^d^Answers that included clinical advice on a specific case and entails communication with a health care professional on the possible benefits and hazards of one or more courses of action.

## Discussion

General practice and community pharmacies are the main sources for questions about CAM to RELIS. This is not surprising since everyone who is resident in a Norwegian municipality is entitled to be registered as a patient with a general practitioner (GP). Drugs prescribed by these GPs are usually provided by local pharmacies. Both GPs and pharmacists are common resources in the primary health care system to discuss CAM with the patients.

Physicians are concerned about adverse effects while pharmacists are concerned about interactions according to our results. We speculate if this could be explained by the different roles of the respective occupations. Physicians focus mainly on diagnosis and treatment of existing diseases and prevention of new when patients use CAM. All questions concerning adverse effects from physicians (n = 12) were all based on symptoms and clinical observations in patients. The questions concerned effects on internal organs (kidney, liver), abnormal laboratory values (liver enzymes, creatinine, sodium, ferritin, cholesterol) and specific central nervous (syncope, seizures), skin (flushing, bruises, hematomas) and gastrointestinal (stomach pain) symptoms. Pharmacists focus on potential interactions with drugs. They are familiar with drug interaction alert systems in the pharmacies to check prescriptions and communicate about this to patients and physicians [13]. A Canadian study found that both consumers and pharmacists thought pharmacists should be knowledgeable about natural health products and able to manage possible drug interactions. Pharmacists in the study tended to place an emphasis on ensuring patient safety, especially with respect to potential interactions with natural health products, as their first priority in patient care [14]. In our results questions about interactions frequently involved drugs with low therapeutic index known to be sensitive to interactions like warfarin and lithium as well as interactions involving other cardiovascular and psychotropic drugs.

About 30% of the 100 answers lacked relevant information to provide factual or consultative replies irrespective if they were patient-specific or general. Questions about documentation of effect and benefit of CAM were the most frequent, and also the category with the highest proportion of answers where relevant information was found (excluding questions about contraindications). Lack of information in answers to questions about interactions reflects the general lack of safety data associated with CAM [15,16]. Although potential benefits of CAM are frequently described in monographs, lack of systematic studies of CAM-drug interactions, in particular for CAM not classified as drugs by the Norwegian Medicines Agency, represent a challenge. The finding that 22% of advanced searches did not retrieve relevant references supports this notion. Relevant references do not include in vitro data, animal studies and retrospective clinical data like case reports because observations in a single patient cannot be generalised to clinical practice.

RELIS include a staff of pharmacists and clinical pharmacologists with training and expertise in literature search and with access to several reference databases [1]. A high proportion of questions to RELIS requires consultative replies, and the competence among the staff and access to several literature sources makes us a valued provider of drug information according to evaluation studies of our service [17,18]. Lack of available relevant information in the case of questions about CAM represents however a medicines information problem, and given the high proportion of patient-specific questions also clinically relevant safety problem. Furthermore, questions about CAM could increase the time spent handling a query, as extensive literature searches may have to be done to make sure no information is overlooked [2].

There were few questions about pregnancy and breastfeeding while this represents about 19% of questions to RELIS concerning drugs. Others have found that many pregnant women use CAM, but do not discuss this with health care professionals. In a study from UK more than 75% of 334 pregnant women did not discuss use of CAM with their physicians or midwives [19]. Furthermore, in a Norwegian study including 600 pregnant women with about 40% using herbal drugs, 8 of 10 women did this based on recommendations from persons other than health care personnel [20]. This could in part be related to the lack of safety information in CAM products over the counter in pharmacies or available from other sources [21]. Lack of safety information could be perceived as “without any risk” by pregnant and breastfeeding women, and reduce the motivation for discussions about safety with health care professionals and thereby RELIS.

### Limitations

This represent a pilot study with a randomly selected sample of 100 QAPs from the RELIS database, about 10% of all QAPs about CAM received in the study period. During the last decade, the proportion of questions involving complementary and alternative medicine (CAM) to RELIS has been about 8%. In 2011, 177 questions (7%) among 2565 involved CAM compared to 157 questions (6%) among 2586 in 2012. There is no reason to believe that variables connected to questions or respective answers in these two years are any different from previous years. Furthermore, a randomly selected sample across a six year period (2005-10) was used in the study to reduce the impact of a single year.

The enquirers represent a selected group of health care professionals, and may not be representative of the general population of their respective occupations. Most questions about CAM are probably not forwarded to health professionals, some are handled by health care professionals themselves, and a limited number of these are forwarded to RELIS. The failure of patients to discuss use of CAM with health care professionals further limits the number and variation of questions we receive. Thus, the results cannot be used to assess frequency of questions about or problems associated with use of CAM. However, they can be used to describe the type of questions submitted by health care professionals. Importantly, the results reflect our experience with how resource demanding questions about CAM can be. This is based on frequent lack of relevant scientific information, a large number of substances in several questions, and a large proportion of patient-specific questions with complex clinical problems. Notice, that in this study, we did not assess the actual advice in each answer, but the information found which could be used to provide it. In RELIS, the staff usually gives advice although no relevant information is found. An example is lack of any safety information in the case of use of CAM during pregnancy where caution is advised.

### Future research

Lack of relevant information to provide answers to questions about CAM represents a particular problem according to our results. A recent study conducted an online search of 13 common herbals with regard to clinical claims, warnings, and other safety information [22]. Less than 3% of 1179 Web sites cited scientific literature to accompany their claims, and this hampers the possibility for clinical decision support. Future studies should assess more in detail the type of questions RELIS and other medicines information centres receive. Furthermore, it would be of great interest to know the implications in clinical practice for the enquirer who has to make a decision.

## Conclusions

This study showed that physicians and pharmacists in general practice and community pharmacies are the main sources for questions about CAM to RELIS. Physicians are concerned about adverse effects while pharmacists are concerned about interactions. The motivation to contact RELIS was based on clinically relevant symptoms and diagnostic observations as well as use of potentially toxic drugs sensitive to interactions. Lack of relevant information to provide answers represents a problem, in particular with regard to the high proportion of complex patient-specific questions to RELIS.

## Abbreviations

CAM: Complementary and alternative medicine; GP: General practitioner; QAP: Question-answer pair; RELIS: Regional Medicines Information and Pharmacovigilance Centres in Norway.

## Competing interests

The authors declare that they have no competing interests.

## Authors’ contributions

HE performed acquisition and analysis of data according to the study protocol, while JS wrote the manuscript. Both authors contributed to conception, design, analysis and interpretation of data. Both authors have been involved in drafting the manuscript and revising it critically for important intellectual content; and have given final approval of the version to be published.

## Pre-publication history

The pre-publication history for this paper can be accessed here:

http://www.biomedcentral.com/1472-6882/14/56/prepub
